# Application of a Zirconium–Titanium Phosphate–Phosphonate Composite Sorbent for Purification of Lithium-Rich Eluate Obtained by Direct Lithium Extraction Technology

**DOI:** 10.3390/ma19173780

**Published:** 2026-09-05

**Authors:** Marta Marszałek, Grzegorz Rotko, Ewa Knapik, Marcin Piotrowski, Kamil Kornaus

**Affiliations:** 1 Cracow University of Technology, Faculty of Chemical Engineering and Technology, Warszawska 24, 31-155 Krakow, Poland; 2 AGH University of Krakow, Faculty of Drilling, Oil and Gas, al. Mickiewicza 30, 30-059 Krakow, Poland; 3 AGH University of Krakow, Faculty of Material Science and Ceramics, al. Mickiewicza 30, 30-059 Krakow, Poland

**Keywords:** direct lithium extraction, lithium-rich eluate, purification, composite sorbent, zirconium phosphonate, titanium phosphate, sorption, oilfield brine

## Abstract

Direct lithium extraction (DLE) represents an emerging technology with considerable potential for lithium recovery from low-grade brines. Nonetheless, DLE still faces several unresolved challenges, particularly concerning the purity of the resulting lithium eluates. The DLE eluate obtained in this study contained approximately 3600 mg/L Li, 2350 mg/L Ca and 1200 mg/L Na. Sorbent-based purification is regarded as one of the most efficient methods for eluate treatment. Among sorbents, titanium phosphates, zirconium phosphates, and zirconium aminotris (methylenephosphonates) are known for their high sorption capacities toward various metal ions. Therefore, this study aimed to develop a zirconium–titanium phosphate–phosphonate sorbent capable of removing impurities from DLE eluates. As a result, a novel powdered ZrTiPO_4_-ATMP sorbent was synthesized under mild conditions. Subsequently, a granular composite sorbent, ZrTiPO_4_-ATMP@PVDF, was prepared using PVDF as a binder. The obtained materials were characterized using XRD, FTIR, SEM-EDS, CHN analysis, and BET analysis. The sorption performance of the composite material was evaluated during the purification of eluates obtained from real oilfield brine. Under optimal conditions, calcium and magnesium removal efficiencies exceeded 95%, while sodium removal reached 60%. At the same time, lithium losses remained below 20%. These losses can be further minimized by returning the regeneration solution to the sorption step.

## 1. Introduction

Lithium-based energy storage, primarily through lithium-ion batteries, is a dominant technology for various applications, including portable electronics, electric vehicles, and grid-scale energy storage [1,2,3]. Lithium is a critical raw material [4], with its extraction concentrated in a few global regions (e.g., South America, Australia, China) [3]. This geographical concentration, combined with rapidly growing demand (it is estimated that the global demand for lithium will increase by 353% between 2024 and 2040) [5], creates significant supply security concerns (in 2025, China, Australia, and Chile accounted for 72% of global lithium mining production) [5]. As the transition to clean energy accelerates, especially with the expansion of electromobility and large-scale energy storage systems [2], the need to secure sustainable and reliable lithium sources becomes urgent [4,6].

Traditional lithium extraction methods, largely reliant on rich mineral deposits (hard-rock pegmatites) and high-lithium brines (salt lake brines), face growing limitations [3,7,8]. These conventional approaches are often environmentally intensive [7,8,9], with high water and energy consumption (80 m^3^ of water and 223 GJ of energy per ton of Li_2_CO_3_ for pyrometallurgical extraction of lithium from spodumene, and 500 m^3^ of water and 40 MJ of energy per ton of Li_2_CO_3_ for evaporation-crystallization extraction of lithium from continental brines) [9], and they cause significant ecological disturbances (e.g., surface degradation, groundwater depletion, biodiversity loss) [7,8,9,10]. Moreover, the finite nature of natural resources, coupled with their uneven geographical distribution, limits their ability to meet future demand. Therefore, relying solely on primary raw materials and conventional methods will not be sufficient to support the rapid scale-up of energy storage technologies [6,7,8,9].

Taking the above considerations into account, the diversification of lithium supply is becoming increasingly critical. One possible solution is the utilization of alternative lithium sources, including various types of brines: oilfield [11,12,13,14,15], geothermal [11,14,16,17], and seawater [11,17,18], with lower lithium concentrations (<50–250 mg/L Li) but greater availability [11,12,13,14,15,16,17,18]. Exploiting these non-conventional resources requires innovative extraction technologies capable of operating efficiently under challenging conditions (e.g., presence of competing ions, lower lithium concentration, brine variability) [11,12,13,14,15,16,17,18].

Among emerging technologies, direct lithium extraction (DLE) has demonstrated significant potential [7,8,9,11,12,19,20,21]. The main DLE methods include adsorption, ion exchange, solvent extraction, membrane-based processes, and electrochemical processes, all of which are designed to selectively and effectively extract lithium ions directly from brine solutions [7,8,19,20,21]. These methods enable lithium recovery from low-grade brines with high efficiency (often above 90% depending on the method used and brine type) [19,20,21] and a reduced environmental footprint, e.g., reduced water consumption compared to evaporation ponds and conventional mining [7,8,9]. Moreover, DLE technologies offer faster lithium recovery, reducing the brine-to-product time from 12–18 months to approximately 24 h [9]. The flexibility of DLE facilitates local production from diverse raw materials, potentially enhancing supply security and supporting the growth of the lithium-ion battery market. Consequently, DLE is positioned as a key technology for diversifying lithium supply chains, improving resource efficiency, and supporting the rapid scale-up of lithium-ion battery production required by the expanding energy storage and electric vehicle sectors [8,20,21]. According to a Goldman Sachs analysis [22], large-scale implementation of DLE could nearly double lithium recovery from brine, improve project economics, and expand global supply by up to 8% by the end of the decade [22].

The most mature and extensively developed DLE technology is sorption (adsorption and ion-exchange) based on specialized materials [20,21]. Lithium-aluminum layered double hydroxides (LiAl-LDHs) are among the key adsorbents. They exhibit high efficiency and are widely employed in industrial-scale applications [8,20,21]. Two prominent classes of ion exchangers (lithium ion sieves) include lithium manganese oxides (LMO) and lithium titanium oxides (LTO) [8,20,21]. These metal oxide-based sorbents operate by reversibly binding lithium ions onto their surfaces, enabling selective extraction even from brines with relatively low lithium concentrations (100–200 mg/L) [21,23,24].

The sorption mechanisms employed by LMO and LTO involve ion exchange and redox reactions (in the case of LMO), allowing for high selectivity toward lithium over competing ions such as magnesium and calcium. This selectivity is crucial for producing concentrates suitable for battery-grade lithium chemicals. Additionally, these materials can be regenerated and reused through controlled desorption processes (up to 20 cycles without significant performance loss in the case of LTO), supporting sustainable and cost-effective operation [24]. LTO sorbents are regarded as more promising lithium-ion sieves due to their superior structural stability and chemical durability, which prevent manganese leaching problems that often limit the lifespan and performance of LMO sorbents [21,23,24].

However, DLE is still an emerging technology with unresolved challenges [9,20], particularly regarding the purity of the lithium eluates produced and the efficient removal of impurities such as magnesium and calcium. Typical calcium and magnesium concentrations in DLE eluates before final purification range from 400 to 4000 mg/L and from 50 to 700 mg/L, respectively [25,26]. The concentrations of these impurities are critical parameters determining the quality of battery-grade lithium compounds and must therefore be reduced to meet battery-grade specifications [27,28]. For lithium carbonate, the maximum allowable impurity concentrations are 250 ppm for Na, 80 ppm for Mg, 50 ppm for Ca, and 10 ppm for K [27]. Even stricter limits apply to lithium hydroxide, where the maximum allowable concentrations are 50 ppm for Na, 10 ppm for Mg, 20 ppm for Ca, and 30 ppm for K [28].

Improving purification methods for lithium concentrates is essential not only to meet the strict quality standards for battery manufacturing but also to enable the large-scale deployment of energy storage technologies. Without reliable and efficient purification, the full potential of DLE and the broader lithium supply chain cannot be realized, thereby limiting progress towards a sustainable energy future [29,30].

Conventional purification methods typically involve multistage processes [31,32,33,34], including chemical precipitation [33], solvent extraction [25,32,33,34], electrodialysis [32], and pressure-driven membrane techniques [32,33], all of which are widely used to reduce impurity levels in lithium concentrates. However, these approaches tend to be resource-intensive, requiring substantial quantities of reagents and generating considerable secondary waste streams, such as significant volumes of sludge or chemical residues per ton of purified lithium.

In response to the aforementioned challenges, we initiated research aimed at developing a method for purifying eluates generated during sorption-based DLE (using an LTO material). The proposed method relies on the application of phosphate and phosphonate sorbents. For this purpose, we selected a group of amorphous sorbents, including titanium phosphates, zirconium phosphates and zirconium aminotris (methylenephosphonates), that had previously been evaluated for copper sorption [35,36]. This choice was motivated by the rapid sorption kinetics exhibited by these materials and by our earlier findings demonstrating their ability to effectively bind calcium and magnesium ions [35,36]. Moreover, these sorbents exhibit a moderate affinity for lithium [37]. The available literature indicates that materials containing organophosphonate ligands and phosphate groups show promising sorption properties and have also been employed for the removal or recovery of lanthanides [38,39,40,41], heavy metals [35,36,42,43,44,45], radionuclides [46,47,48], as well as cesium [49,50], potassium [49], and strontium [50]. Therefore, we hypothesized that these materials could be effectively applied in the polishing treatment of DLE eluates.

The objective of this work was to develop a novel zirconium–titanium phosphate–phosphonate composite sorbent for the effective removal of impurities (mainly magnesium, calcium, sodium and potassium) from DLE eluates. To this end, a screening study was conducted to identify promising candidates among phosphate- and phosphonate-based materials for potential application in DLE eluate purification. The screening results identified two best-performing materials: amorphous titanium phosphate and amorphous zirconium phosphonate. Based on the complementary properties of these two materials, a new powdered sorbent containing both zirconium and titanium, as well as phosphate and phosphonate groups, was synthesized under mild conditions. Subsequently, a composite sorbent in granular form was prepared using PVDF as a binder. The synthesized materials were characterized using X-ray diffraction (XRD), Fourier transform infrared (FTIR) spectroscopy, scanning electron microscopy coupled with energy-dispersive X-ray spectroscopy (SEM-EDS), CHN elemental analysis, mercury intrusion porosimetry (MIP), and Brunauer–Emmett–Teller (BET) analysis. The sorption performance of the powdered and composite materials was evaluated using both a model solution and a DLE eluate obtained from real oilfield brine treated with a hydrogen titanium oxide (HTO) ion sieve.

The novelty of this work lies in the development of a new zirconium–titanium phosphate–phosphonate sorbent composition and its application to the purification of lithium-rich eluates generated by sorption-based DLE. To the best of our knowledge, zirconium–titanium phosphate–phosphonate sorbents have not previously been reported for the purification of DLE eluates. In addition, the preparation of the material in granular form using PVDF as a binder provides a practically relevant formulation that facilitates handling and its potential application in sorption processes.

## 2. Materials and Methods

### 2.1. Materials

The following reagents were used in this study: 65% nitric acid, 85% phosphoric acid, 37% hydrochloric acid, 25% ammonia solution, sodium chloride, anhydrous calcium chloride, magnesium chloride hexahydrate, 96% ethyl alcohol, and N,N-dimethylformamide (DMF), all obtained from Chempur (Piekary Śląskie, Poland); titanium (IV) oxide sulfate sulfuric acid hydrate, zirconium carbonate basic hydrate and powdered poly (vinylidene fluoride) (PVDF) purchased from Alfa Aesar (Ward Hill, MA, USA); titanium dioxide (anatase), lithium carbonate, anhydrous lithium chloride and aminotris (methylenephosphonic acid) solution 50% in water (ATMP) provided by Sigma Aldrich (St. Louis, MO, USA); and lithium hydroxide monohydrate delivered by Krakchemia S.A. (Kraków, Poland).

In the preliminary sorption tests, a commercially available chelating resin, Puromet^TM^ MTS9500 (a polystyrenic, microporous aminophosphonic resin designed for iron, copper, zinc, antimony and bismuth removal with a calcium capacity of 26 g/L) [51] obtained from Purolite (King of Prussia, PA, USA), as well as previously synthesized sorbents, were used. The latter include three titanium phosphates (TiP3, TiP4, and TiP5) and three zirconium phosphates (ZrP3, ZrP4, and ZrP5), the synthesis and physicochemical characterization of which were comprehensively described in our earlier work [35], as well as six zirconium phosphonates (Zr-ATMP3, Zr-ATMP4, Zr-ATMP5, Zr-ATMP6, Zr-ATMP7, and Zr-ATMP8), whose synthesis and characterization were reported in detail in another of our previous studies [36].

**Figure 1:**
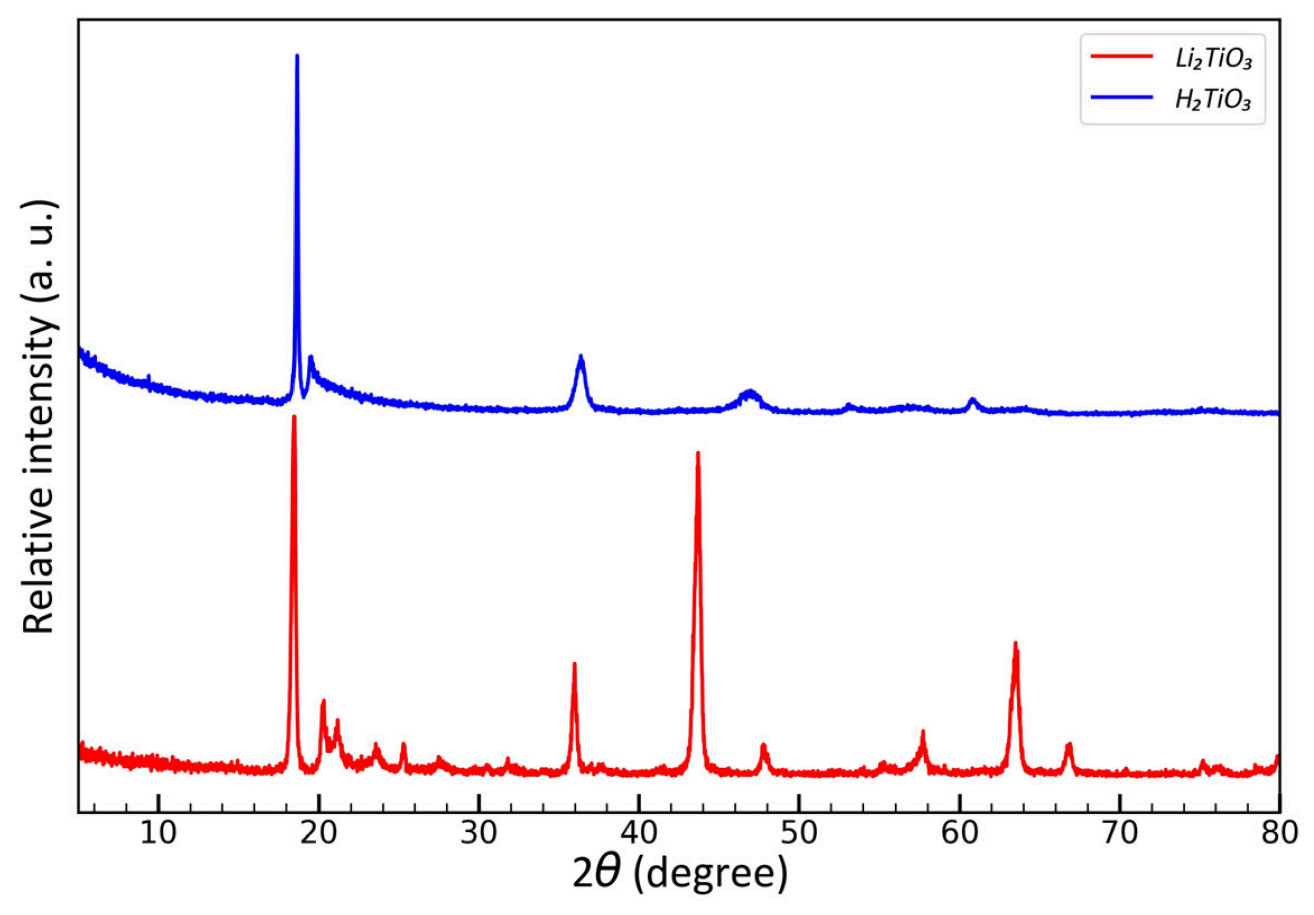
XRD patterns of Li_2_TiO_3_ and H_2_TiO_3_.

To obtain lithium-rich eluate from the DLE process, a hydrogen titanium oxide ion sieve synthesized according to the procedure described in [52] and lithium-rich oilfield brine were used. The synthesis of the HTO sorbent involved two steps. In the first step, Li_2_TiO_3_ was obtained by a solid-state reaction of titanium dioxide (anatase) with lithium carbonate at a calcination temperature of 700 °C. The reactants were mixed in such a way as to achieve a lithium-to-titanium molar ratio of 2:1. In the second step, lithium was eluted from the Li_2_TiO_3_ using a hydrochloric acid solution to obtain H_2_TiO_3_. The purity of the materials (Li_2_TiO_3_ and H_2_TiO_3_) was verified by the XRD method (Figure 1).

Lithium-rich brine was collected from a separation tank at a surface facility in one of the oilfields located in the Polish Lowlands in northwestern Poland. The sample was taken at a pressure of approximately 3 bar, while avoiding aeration, and was analyzed within 24 h in accordance with the standardized procedures described in [53]. The collected brine did not contain any visible impurities (floating hydrocarbon layers or sediment). For sorption experiments, the brine was used without any additional treatment, except for prior filtration through a polypropylene bag filter with a nominal pore size of 5 µm delivered by Techniczne24 (Stalowa Wola, Poland).

### 2.2. Preparing a Lithium-Rich Eluate

The lithium-rich eluate was prepared using two methods.

Method 1: 20 g of H_2_TiO_3_ sorbent was mixed with 1 kg of oilfield brine, and subsequently, 70 mL of 1 M NaOH was added in several portions. The pH of the mixture was in the range of 7.0–7.5. The suspension was then stirred for at least 5 h and allowed to settle. After settling, the oilfield brine was decanted, and the sorbent was washed four times with deionized water (each portion of water was 200 mL). Thereafter, the washed sorbent was treated with 0.1 M HCl (200 mL added in several portions) and stirred for at least 5 h, followed by settling. The clear eluate was collected, and the desorption procedure was repeated three more times, resulting in a total of four fractions.

Method 2: 20 g of H_2_TiO_3_ sorbent was mixed with 1 kg of oilfield brine, and subsequently, 70 mL of 1 M NaOH was added in several portions. The pH of the mixture was in the range of 7.0–7.5. The resulting suspension was stirred for at least 5 h and then left undisturbed to allow the sorbent to settle. Once phase separation had occurred, the oilfield brine was decanted, and the sorbent was washed four times with deionized water (200 mL per wash). In the desorption step, 50 mL of deionized water was added to the washed sorbent, followed by the dropwise addition of 50 mL of 1 M HCl. The resulting suspension was stirred for 5 h and then left to settle. After phase separation, the eluate (pH ≈ 1.5) was collected, and the sorbent was subsequently contacted with a fresh portion of oilfield brine (1 kg), to which 70 mL of 1 M NaOH was gradually added. The obtained suspension was stirred for at least 5 h and then left to allow the sorbent to settle. After settling, the oilfield brine was decanted, and the sorbent was washed with deionized water in the same manner as earlier. In the next step, the sorbent was subjected to desorption by treating it with the eluate from the previous stage, to which 15 mL of 3 M HCl was added. The suspension was stirred for at least 5 h and allowed to settle. After phase separation, the eluate was collected, and the sorption-washing-desorption procedure was repeated two more times (using the eluate and 3 M HCl for desorption), resulting in the final lithium-rich eluate.

### 2.3. Synthesis of Powdered Zirconium–Titanium Phosphate–Phosphonate Sorbent Material

The synthesis of the powdered zirconium–titanium phosphate–phosphonate sorbent material (named ZrTiPO_4_-ATMP) was carried out using the precipitation method under mild conditions, specifically at ambient temperature and atmospheric pressure. Initially, a zirconyl nitrate solution was obtained by reacting 33.3 g of zirconium carbonate basic hydrate with 35 mL of 65% nitric acid. The resulting solution was then diluted with 200 mL of 1.0 M nitric acid. Subsequently, a titanyl sulfate solution was prepared by dissolving 24.2 g of titanium (IV) oxide sulfate sulfuric acid hydrate in 320 mL of a 1.0 M nitric acid solution. The next step involved the preparation of a mixture containing phosphoric acid and aminotris (methylenephosphonic acid). To achieve this, 250 g of a 50% ATMP solution, 142 g of an 85% phosphoric acid solution, and 392 g of deionized water were mixed. While stirring the prepared mixture continuously, zirconyl nitrate and titanyl sulfate solutions were added dropwise. After the precipitation was complete, the mixture was stirred for an additional 1.5 h and then left for 48 h to allow the formed precipitate to age. After this period, the mixture was separated by centrifugation. The obtained precipitate was washed with deionized water until the pH of the washings reached approximately 4, after which it was dried at 60 °C. Finally, the sorbent was ground into a powder.

### 2.4. Synthesis of Zirconium–Titanium Phosphate–Phosphonate Sorbent Composite Material

A zirconium–titanium phosphate–phosphonate sorbent composite material (ZrTiPO_4_-ATMP@PVDF) was prepared via the phase inversion method [54] using PVDF as a binder. The preparation method used herein is a modification of the procedure reported by Park et al. [55] for the scalable preparation of MOF beads. A total of 5 g of PVDF powder was dissolved in 54 mL of N,N-dimethylformamide under stirring at 60 °C for 3 h. Subsequently, 10 g of ZrTiPO_4_-ATMP powder was dispersed into the PVDF solution. The resulting slurry was transferred into a syringe and vertically dropped into a nonsolvent solution (water/ethyl alcohol, 5:1, *v*/*v*). Upon contact with the solution, solid beads precipitated immediately, driven by solvent–water exchange. The beads were mixed in the bath for 10 min, after which the solution was decanted. Then, they were washed three times with water (500 mL; mixing time = 30 min) and stored moist (prior to use, the water content was measured using a moisture analyzer). For characterization, a sample of the beads was dried at 60 °C for 12 h.

### 2.5. Sorption Tests

All sorption experiments were performed at room temperature using the batch contact method. Preliminary sorption and kinetic tests of selected sorbents were performed using a solution with a composition corresponding to that of the eluate obtained by Method 1. Because a large volume of solution was required for the sorption tests, a simulated solution was prepared. The solution was obtained by dissolving appropriate amounts of metal salts (sodium chloride, anhydrous lithium chloride, anhydrous calcium chloride, and magnesium chloride hexahydrate) in deionized water. The composition of the solution was as follows: 495 mg/L Li, 55 mg/L Na, 141 mg/L Mg, and 331 mg/L Ca.

In the preliminary tests, 0.5 g of the selected sorbent or resin was added to 50 mL of the solution, shaken vigorously for 1 h and then left undisturbed for an additional 23 h (the total contact time was 24 h). After the desired contact time, the mixture was centrifuged to separate the solid and liquid phases, and the supernatant was subsequently analyzed using atomic absorption spectrometry (AAS). Two series of tests were performed: one without pH adjustment (pH of approximately 2) and one with the pH adjusted to 8. The pH correction was carried out using concentrated ammonia solution dispensed with a micropipette. The pH was monitored using a CX-701 multifunction meter (ELMETRON, Zabrze, Poland). Each series was performed in triplicate, and the results were averaged.

Sorption kinetics experiments were carried out using the same quantities of sorbent and solution as those used in the preliminary studies, in order to ensure consistency of the experimental conditions. Samples of the sorbent–solution mixture were collected at the following contact times: 1 min, 5 min, 10 min, 15 min, 30 min, 45 min, 60 min, and 24 h. Immediately after sampling, the sorbent was separated from the solution by centrifugation. The resulting supernatant was subsequently subjected to quantitative analysis of residual metal ion concentrations using the AAS method. All kinetic experiments were performed in triplicate, and the mean values were used for further analysis.

The removal efficiency of a given metal *R_Me_* (%) was calculated according to Equation (1):

(1) $$R_{Me}=\frac{C_{0}-C_{t}}{C_{0}}\cdot 100\%$$

where *C*_0_ (mg/L) is the initial concentration of a given metal, and *C_t_* (mg/L) is the concentration of a given metal after a specific contact time between the sorbent and the solution.

Sorption experiments evaluating the use of the ZrTiPO_4_-ATMP@PVDF composite for the purification of DLE eluate were performed using eluate obtained according to Method 2. Two series of sorption experiments were carried out: the first investigated the effect of adding different amounts of LiOH as a pH-adjusting agent, while the second examined the effect of the sorbent mass-to-solution volume ratio. The first series of experiments was performed as follows: 4 g of sorbent beads and 20 g of eluate were mixed for 1 h with the appropriate addition of 2 M LiOH solution (0.69, 1.73, 2.76, 4.14, and 5.52 mL). The selected volumes of 2 M LiOH solution corresponded to 10%, 25%, 40%, 60%, and 80% of the OH^−^ ion demand, as determined based on the ion exchange stoichiometry (taking into account the total amount of cations present in the eluate). The pH of the mixture was monitored throughout the experiment. In the second series of experiments, 5 g of sorbent beads was mixed with varying volumes of eluate (20, 40, 60, 80 and 100 mL) and a fixed volume of 1.73 mL of 2 M LiOH solution. The contact time between the sorbent beads and the eluate was set to 1 h in all cases. Upon completion of the contact period, the sorbent beads were separated from the eluate, and the residual concentrations of metal ions in the eluate were determined using the AAS method. Both sorption experiments were repeated three times, and the results were averaged.

### 2.6. Regeneration Tests

Regeneration tests were performed using ZrTiPO4-ATMP@PVDF composite beads loaded under optimal sorption conditions (5 g of sorbent, 1.73 mL of 2 M LiOH, and 80 mL of eluate). For each test, 5 g of the loaded sorbent was treated with 15 mL of HCl solution at varying concentrations (1 M, 2 M and 4 M). The contact time was 1 h. The concentrations of metal ions in the solutions after desorption were determined using the AAS method. All treatments were conducted in triplicate, and the results were averaged.

### 2.7. Characterization and Analytical Methods

X-ray diffraction patterns (2θ scan range: 5–80°) were recorded using a SmartLab SE powder X-ray diffractometer equipped with a HyPix-400 detector (Rigaku, Tokyo, Japan). The X-ray source was a copper anode tube operated at 40 kV and 50 mA. The scan was performed at a speed of 3.00°/min with a step width of 0.02°.

FTIR spectra were recorded using a Nicolet iS5 FTIR spectrometer equipped with an iD7 attenuated total reflection (ATR) accessory with a diamond crystal (Thermo Fisher Scientific, Waltham, MA, USA). The spectra were collected in the range of 400–4000 cm^−1^ with a total of 32 scans.

Scanning electron microscope images were acquired using an Apreo 2 S LoVac high-resolution scanning electron microscope (Thermo Fisher Scientific, Waltham, MA, USA) at an acceleration of 1.5 kV and magnifications of 50,000× and 100,000×. The surface elemental composition (in mapping mode) was determined using an UltraDry EDS detector (Thermo Fisher Scientific, Waltham, MA, USA). Two accelerating voltage values, 15 kV and 25 kV, were used during the SEM-EDS analysis. The samples were mounted on conductive carbon tape and sputter-coated with a thin layer of gold.

CHN elemental analysis was performed using a Perkin Elmer 2400 Series II CHN Elemental Analyzer (PerkinElmer, Waltham, MA, USA). The combustion temperature was 925 °C, while the reduction temperature was 640 °C.

Mercury intrusion porosimetry was used to characterize the pore structure of the samples. Measurements were carried out using a PoreMaster 33 mercury porosimeter (Quantachrome Instruments, Boynton Beach, FL, USA) over a pressure range from 2.5 psi to 33,000 psi, corresponding to pore diameters ranging from approximately 100 µm to 3 nm, based on the Washburn equation:

(2) $$D=\frac{-4\gamma \cos\theta }{P}$$

where *D* is the pore diameter, *γ* is the surface tension of mercury (480 mN/m), *θ* is the contact angle between mercury and the solid (assumed to be 140°), and *P* is the applied pressure.

Before analysis, samples were dried under vacuum to remove moisture and volatiles that could interfere with mercury intrusion. The measurements were conducted using a two-stage process: a low-pressure stage (2.5–50 psi) and a high-pressure stage (20–33,000 psi). Key parameters such as total intruded volume, porosity, apparent and skeletal density, and pore size distribution were obtained directly from the instrument’s software.

Nitrogen sorption measurements at low temperature were performed using the volumetric method in a discontinuous manner with a Micromeritics ASAP 2020 analyzer (Micromeritics Instrument Corporation, Norcross, GA, USA). The sample was degassed at 130 °C for 24 h under a vacuum of 10^−3^ mmHg. The Brunauer–Emmett–Teller (BET) multipoint method was used to determine the specific surface area at relative pressures (*p/p_0_*) ranging from 0.05 to 0.25. Dead volume calibration was performed by the apparatus before the actual measurement. Nitrogen with a purity of 99.999% was used as the adsorbate, and the measurements were conducted at the temperature of liquid nitrogen (77 K). The distribution of mesopore sizes was determined using the Barrett-Joyner-Halenda (BJH) model, and DFT (density functional theory) analyses did not reveal the presence of micropores.

The concentrations of Li, Na, K, Mg, and Ca in the solutions were determined using a PerkinElmer 1100B atomic absorption spectrometer (PerkinElmer, Waltham, MA, USA). Each reported value represents the average of three measurements.

The concentrations of Al, Ag, B, Ba, Be, Cd, Co, Cs, Fe, Mn, Ni, Pb, Rb, Sr, Ti, V, and Zr were determined using an Elan DRC-e inductively coupled plasma mass spectrometer (ICP-MS) (PerkinElmer Inc., Waltham, MA, USA). A detailed description of the analysis is provided in [56].

Total dissolved solids (TDS) and total suspended solids (TSS) in raw oilfield brine were determined gravimetrically according to [57]. Turbidity was measured using a DR/2000 spectrophotometer (HACH, Loveland, CO, USA), and pH was determined with a CX-701 multifunction meter (ELMETRON, Zabrze, Poland). The content of dissolved hydrocarbons was quantified as the mineral oil index (sum of C10–C40 hydrocarbons) according to [58], employing an HP 5890 gas chromatograph (Agilent, Wilmington, DE, USA). The chloride content was determined by argentometric titration using the Mohr method [53].

## 3. Results and Discussion

### 3.1. DLE Process—Preparation of Lithium-Rich Eluate

A typical hydrogen titanium oxide ion sieve in powder form was used for direct lithium extraction from oilfield brine. The physicochemical properties and composition of the brine used in the process are presented in Table 1 and Table A1 (see Appendix A). As can be seen, the brine used as a lithium-bearing resource represents a challenging feedstock.

The DLE process was conducted in suspension, and due to the high concentrations of calcium and magnesium during sorption, the pH was adjusted to 7.0–7.5. Exceeding this pH range caused the precipitation of calcium and magnesium hydroxides, which led to a decrease in the quality of the eluate.

The regeneration of the HTO sorbent was carried out in two ways: Method 1—using several portions of diluted hydrochloric acid (0.1 M), and Method 2—by continuously adding portions of 3 M hydrochloric acid solution to the sorbent suspension while maintaining the pH in the range of 1–2. The first method was employed to determine whether it is possible to desorb the ions bound to the sorbent material by mixing it with successive portions of acid. A low acid concentration was selected to minimize the risk of sorbent degradation and to attempt the selective leaching of individual ions. The results of these tests are presented in Table 2.

The first conclusion is that the obtained eluates are characterized by low lithium concentrations. It is evident that, in the first stage, mainly sodium, calcium and magnesium are leached. It should be noted that, after sorption, the sorbent was thoroughly rinsed with deionized water, and the final rinse water contained no more than 30 mg/L of sodium. Therefore, the high sodium concentration observed in the first eluate fraction must be attributed to sodium sorbed onto the HTO. Analysis of the sodium desorption profile (Table 2) indicates that sodium is relatively weakly bound and can be removed easily. It should be emphasized that the majority of the interfering ions were removed during the DLE process. Therefore, the process can be considered highly attractive, even for such a challenging raw material as nearly saturated formation brine. On the other hand, interfering ions, particularly calcium, must be removed from the eluate, as their concentration is comparable to that of lithium. This results from the high calcium concentration in the raw material.

Due to the relatively high solubility of calcium hydroxide (1.73 g/L at 20 °C), it cannot be precipitated by increasing the pH above 10, unlike in the case of magnesium hydroxide. Therefore, we decided to develop a sorption material suitable for this purpose. Preliminary sorption tests were conducted using a solution reflecting the composition of Fraction 3. At the same time, we made efforts to modify the sorbent regeneration process in order to obtain an eluate with the highest possible lithium concentration. To this end, the desorption process was conducted at a constant pH by the gradual addition of 3 M hydrochloric acid. The obtained eluate was recycled in the subsequent desorption cycle (Method 2). All tests were performed using the same sorbent sample, demonstrating the possibility of multiple reuses. The key process results for each cycle are summarized in Table 3 and Table 4, while the cation composition (including trace elements) of the eluate (cycle number 4) is presented in Table A1.

As can be seen, the sorption efficiency decreases with each subsequent cycle. This is due to the incomplete recovery of lithium ions during the regeneration stage. The main reason for this is that the eluate from the previous cycle comes into contact with the sorbent during regeneration, shifting the equilibrium in an unfavourable direction for desorption. The experiment showed that this desorption method enables the production of much more concentrated lithium solutions, but at the expense of reduced desorption efficiency, which in turn negatively affects sorption efficiency. This case should be considered a borderline scenario. In a real process, desorption can be carried out in multiple stages using a mixed approach (a combination of Methods 1 and 2). The aim of this part of the research was to produce lithium-rich eluate samples from real raw material using the most promising types of sorbents in order to determine the range of eluate compositions within which sorbents used for eluate purification should operate. Therefore, no further optimization of the DLE process was carried out in this study. The main conclusion from this part of the work is that the DLE process enables the production of lithium-rich eluates with lithium concentrations exceeding the level required for lithium carbonate precipitation (approximately 1900 mg/L). Moreover, solutions with lithium concentrations above 3000 mg/L can be effectively processed by electrodialysis to obtain lithium hydroxide [29]. However, the resulting concentrate requires further purification, with particular emphasis on the removal of calcium ions. In the case of such highly concentrated solutions, calcium can be partially removed by hydroxide precipitation. The calcium concentration in a saturated hydroxide solution is approximately 900 mg/L. However, in a multicomponent solution, this value may vary. Precipitation using calcium carbonate is also effective, but in this case, it may lead to the co-precipitation of lithium carbonate. For these reasons, the possibility of calcium removal using sorbents was investigated.

It should be noted that contamination with calcium and magnesium hinders both the production of lithium carbonate by precipitation and the production of LiOH by electrodialysis (membrane degradation) [33]. Contamination with sodium and potassium does not pose a problem during lithium carbonate precipitation, but it adversely affects electrodialysis, resulting in lithium hydroxide of lower purity [33].

### 3.2. Preliminary Sorption Tests—Sorbent Selection

Following the motivation outlined in the Introduction, attention was focused on phosphate and phosphonate materials due to their high sorption capacities and favourable sorption kinetics. Preliminary tests were performed using a solution with a composition corresponding to the eluate obtained by Method 1. Static tests were carried out using the solution both without pH adjustment and with pH adjusted to 8. The aim of these tests was to identify materials with the most favourable sorption properties. The removal efficiencies of Ca, Na, Mg, and Li were used as the selection criteria. Potassium concentration measurements were omitted due to the low potassium content in this type of eluate. Twelve previously synthesized phosphate and phosphonate materials were tested: three titanium phosphates (TiP3, TiP4, and TiP5), three zirconium phosphates (ZrP3, ZrP4, and ZrP5), and six zirconium phosphonates (Zr-ATMP3, Zr-ATMP4, Zr-ATMP5, Zr-ATMP6, Zr-ATMP7, and Zr-ATMP8). To compare the properties of phosphate and phosphonate sorbents with those of commercial materials, a commercially available resin (Puromet^TM^ MTS9500 with a calcium capacity of 26 g/L) was also tested. The results are presented in Table 5.

Materials synthesized at high phosphoric acid-to-metal or ATMP-to-metal molar ratios were selected: TiP3 (P:Ti = 2:1), TiP4 (P:Ti = 5:1), TiP5 (P:Ti = 10:1), ZrP3 (P:Zr = 2:1), ZrP4 (P:Zr = 5:1), ZrP5 (P:Zr = 10:1), Zr-ATMP3 (P:Zr = 2:1), Zr-ATMP4 (P:Zr = 5:1), Zr-ATMP5 (P:Zr = 10:1), Zr-ATMP6 (P:Zr = 25:1), Zr-ATMP7 (P:Zr = 50:1), and Zr-ATMP8 (P:Zr = 100:1) due to their superior sorption performance, as indicated by our previous studies [35,36]. Our research [35,36] demonstrated that these sorbents can be used not only for the sorption of d-block metals but also for magnesium and calcium. The results of this work (Table 5) show that, at acidic pH, the removal of contaminants is moderate, but some trends are evident: phosphate materials exhibit higher sodium removal efficiencies, Zr-ATMP sorbents (except Zr-ATMP3) exhibit significantly higher removal efficiencies for magnesium and calcium, and in all cases, lithium losses are minimal.

Moreover, almost all Zr-ATMP materials exhibit higher calcium removal efficiencies than the reference resin Puromet^TM^ MTS9500. Lithium losses are significantly lower for both phosphonate and phosphate materials than for Puromet^TM^ MTS9500. The sodium removal efficiency of the resin was not assessed, as the resin still contained some sodium even after washing with hydrochloric acid. These ions were exchanged during the process, resulting in negative R_Na_ values.

Preliminary results obtained at alkaline pH are significantly better for all materials. The vast majority of phosphate and phosphonate sorbents, as well as the Puromet^TM^ MTS9500 resin, exhibit calcium and magnesium removal efficiencies higher than 90%. A substantial difference is observed in sodium removal: Zr-ATMP sorbents remove almost no sodium, while phosphate sorbents can remove even more than 90% (TiP5). Lithium losses for phosphate and phosphonate materials are also significant (27–53%) and higher than those observed for Puromet^TM^ MTS9500 (26%).

Taking into account the fact that zirconium phosphonate materials are effective for calcium and magnesium removal even at acidic pH, and that titanium phosphate materials are effective in sodium removal, attempts were made to combine both of these beneficial features. First, a physical mixture of the two most effective sorbents, TiP5 and Zr-ATMP7 (designated in Table 5 as TiP5 + Zr-ATMP7), was used. Equal amounts of both sorbents were applied in the sorption experiments. Second, a mixed sorbent containing titanium and zirconium, as well as phosphate and phosphonate groups (ZrTiPO_4_-ATMP), was synthesized. Promising results were observed in both cases:

- Lithium losses at acidic pH are negligible and significantly lower than those observed for the Puromet^TM^ MTS9500 resin, whereas at alkaline pH, lithium losses for the ZrTiPO_4_-ATMP sorbent are comparable to those for the Puromet^TM^ MTS9500 resin;
- Calcium removal at acidic pH is more efficient for both the physical mixture of TiP5 and Zr-ATMP7 and the ZrTiPO_4_-ATMP sorbent than for the Puromet^TM^ MTS9500 resin, whereas at alkaline pH, the removal efficiencies are comparable;
- Magnesium removal at acidic pH is more efficient for the ZrTiPO_4_-ATMP sorbent than for both the physical mixture of TiP5 and Zr-ATMP7 and the Puromet^TM^ MTS9500 resin; at alkaline pH, magnesium is almost completely removed by the ZrTiPO_4_-ATMP sorbent, while the physical mixture of TiP5 and Zr-ATMP7 yields slightly worse results;
- The ability to remove sodium is retained for both the physical mixture of TiP5 and Zr-ATMP7 and the ZrTiPO_4_-ATMP sorbent, although the efficiency is significantly lower than that of TiP5—this is likely related to the content of functional groups responsible for sodium removal in the material.

In view of the overall assessment of sorbent effectiveness, ZrTiPO_4_-ATMP was selected for further testing, as it effectively binds all key impurities while causing minimal lithium loss.

### 3.3. Synthesis and Characterization of Powdered ZrTiPO_4_-ATMP Sorbent Material and ZrTiPO_4_-ATMP@PVDF Composite Sorbent Material

Based on the superior sorption performance of the previously synthesized sorbents Zr-ATMP7 and TiP5 in preliminary tests, a mixed material was developed. The powdered ZrTiPO_4_-ATMP sorbent was prepared under mild conditions using a precipitation method. The material was precipitated by combining a solution of zirconium and titanium ions (1:1 molar ratio) with a 1:1 molar mixture of phosphoric acid and ATMP.

**Figure 2:**
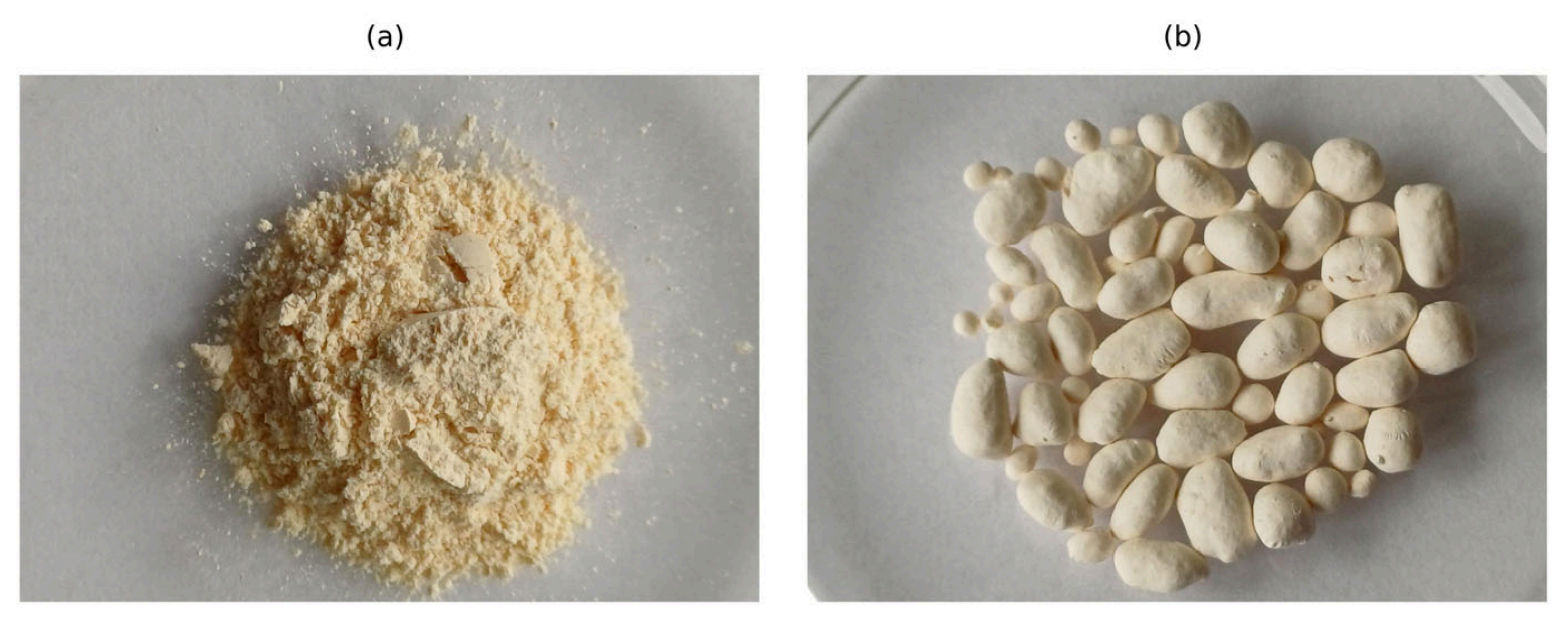
Photographs of (**a**) ZrTiPO_4_-ATMP powder and (**b**) ZrTiPO_4_-ATMP@PVDF granules.

In order to obtain the material in granular form, the phase inversion method was applied, using PVDF as a binder. Spheroidal granules with a diameter of 1–2 mm were obtained. Figure 2 shows images of the ZrTiPO_4_-ATMP sorbent material in powder form (light beige in color) and the ZrTiPO_4_-ATMP@PVDF composite sorbent material in granular form.

**Figure 3:**
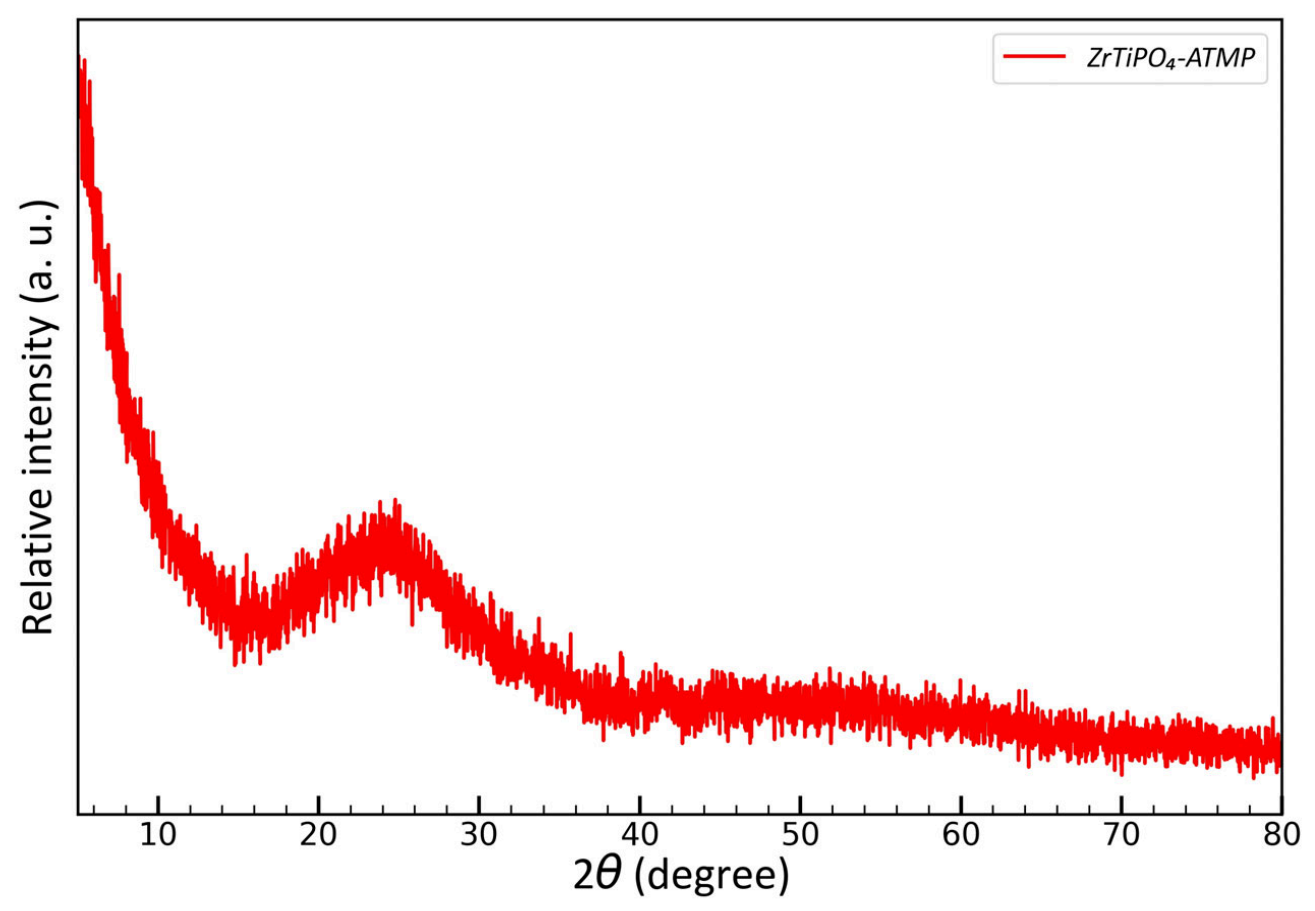
XRD pattern of ZrTiPO_4_-ATMP powder.

Figure 3 presents the X-ray diffraction pattern of the obtained ZrTiPO_4_-ATMP powder. Similar to other previously synthesized zirconium phosphonate and titanium phosphate materials, ZrTiPO_4_-ATMP is completely amorphous, as expected.

**Figure 4:**
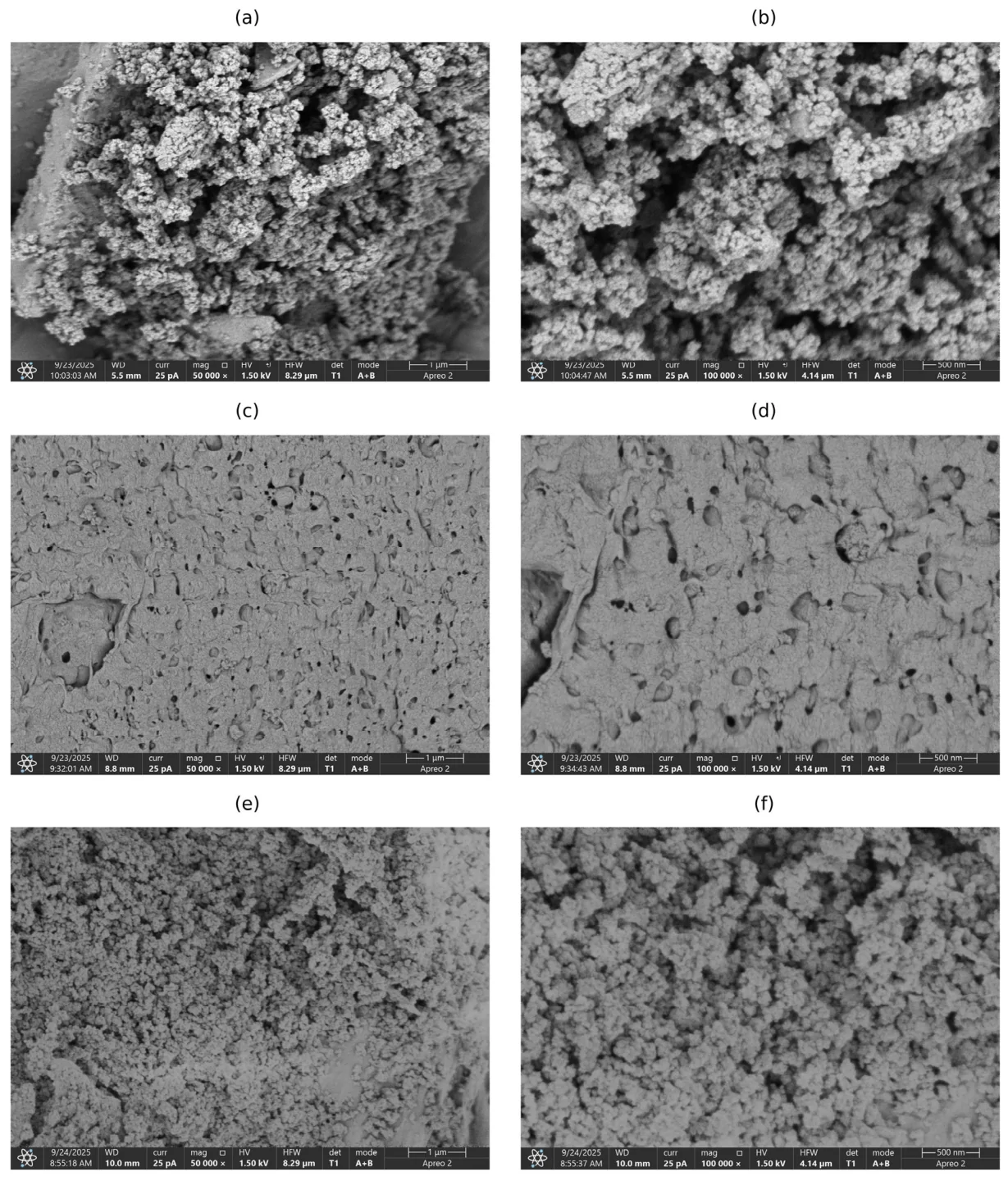
SEM images of the ZrTiPO_4_-ATMP powder sorbent at magnifications of (**a**) 50,000× and (**b**) 100,000×; surface of ZrTiPO_4_-ATMP@PVDF composite beads at magnifications of (**c**) 50,000× and (**d**) 100,000×; cross-section of ZrTiPO_4_-ATMP@PVDF composite beads at magnifications of (**e**) 50,000× and (**f**) 100,000×.

The surface morphology of the ZrTiPO_4_-ATMP powder and ZrTiPO_4_-ATMP@PVDF granules (surface and cross-section) was characterized using a scanning electron microscope. Figure 4 shows SEM images at magnifications of 50,000× and 100,000×.

SEM micrographs of the ZrTiPO_4_-ATMP powder sorbent revealed irregular, amorphous aggregates with a highly branched, bushy morphology (Figure 4a,b). The surface appeared rough and heterogeneous, lacking well-defined crystalline domains, which is typical for materials obtained by precipitation methods under relatively mild conditions. In contrast, alternative synthesis approaches, such as hydrothermal methods, often promote the formation of more ordered, crystalline structures. Importantly, the amorphous, branched morphology observed here is considered beneficial for ion-exchange applications, as it increases the accessibility of active sites and enhances the sorbent’s ion-exchange capacity. Such structural features have previously been reported for zirconium phosphate- and zirconium phosphonate-based sorbents used in the removal of metal ions from aqueous solutions [35,59].

The prepared ZrTiPO_4_-ATMP@PVDF composite beads are spheroidal, with an average diameter of a few millimeters. The external surface of the beads appears smooth upon visual inspection, but at higher magnifications (Figure 4c,d), the material is clearly porous, featuring tortuous channels unevenly distributed across the surface. The phase inversion method, which induces the precipitation of PVDF fibers, is difficult to control, and the expelled solvent forms random, tortuous pores. These pores extend into the interior of the beads rather than merely forming surface irregularities, which is advantageous because ion exchange can occur throughout the entire bead volume. Cross-sectional images of the composite beads (Figure 4e,f) show that the ZrTiPO_4_-ATMP powder is uniformly distributed throughout the beads. Porosity is maintained across the entire cross-section, even near the core. The PVDF content was properly selected, ensuring mechanical stability without excessive accumulation of the binder (PVDF), while the powder retained its original morphology.

The composition of the prepared materials was studied using SEM-EDS (Table 6) and elemental (CHN) analysis (Table 7).

EDS microanalysis of the ZrTiPO_4_-ATMP powder was performed at two different acceleration voltages and revealed significant differences in the sample composition. It should be noted that the electron beam penetration depth depends on the acceleration voltage, as well as on the density of the material and the atomic mass of the elements present in the sample [60]. According to the Kanaya–Okayama model [61], for the same analyzed area (the same material properties), increasing the acceleration voltage from 15 kV to 25 kV more than doubles the penetration depth. Therefore, the results obtained at 25 kV reflect the local composition of a thicker layer of the sorbent, while those recorded at 15 kV provide information on a nearer-surface region.

The content of P, Zr, and Ti is higher in the results recorded at 15 kV. Increasing the acceleration voltage leads to a significant decrease in titanium and zirconium content, accompanied by a significant increase in nitrogen and carbon content. The high oxygen content does not depend on the acceleration voltage. Therefore, it can be concluded that the surface layer of the sorbent powder is richer in zirconium and titanium phosphates, while deeper layers (the core of the sorbent grains) are composed almost exclusively of zirconium phosphonate (Zr-ATMP). The presumed core–shell-like structure may result from differences in the precipitation dynamics of the individual components.

Two EDS measurements were performed on the ZrTiPO_4_-ATMP@PVDF composite granules: one on the surface of a granule and the other on a freshly cut cross-section. The content of the ZrTiPO_4_-ATMP sorbent material on the surface of the granule is low. The main component detected is fluorine, originating from PVDF. Therefore, it can be concluded that during the forming process, PVDF forms a kind of membrane on the granule surface. This is consistent with the SEM imaging results, which show a relatively smooth surface. The fluorine content in the cross-section is significantly lower, while the contents of P, Zr, Ti, and N are significantly higher, confirming that the ZrTiPO_4_-ATMP material is located inside the bead and surrounded by the polymer.

The total C, H, and N contents in both samples are low due to the fact that the main components of the powder sorbent are phosphorus, oxygen, zirconium and titanium. Additionally, PVDF, which was added as a binder, contains almost 60 wt.% fluorine. Considering that the ZrTiPO_4_-ATMP:PVDF ratio of 2:1 was used in the granulation process, the expected C, H, and N contents in the composite should be 16.85 wt.%, 3.08 wt.%, and 1.65 wt.%, respectively. These observed discrepancies may result from the fact that some of the DMF used as a solvent could have remained trapped in the polymer matrix and been difficult to remove. The C:N molar ratio in the ZrTiPO_4_-ATMP sorbent is 3.1, which is in good agreement with the theoretical value of 3 for the ATMP molecule, assuming that all carbon and nitrogen atoms originate solely from ATMP. The H:C molar ratio in the ZrTiPO_4_-ATMP material is 5.4, which is higher than expected if hydrogen originated only from ATMP moieties (the theoretical value is ca. 3.3). The most likely explanation is that the excess hydrogen originates from hydrogen phosphate groups and constitutive water present in the material.

**Figure 5:**
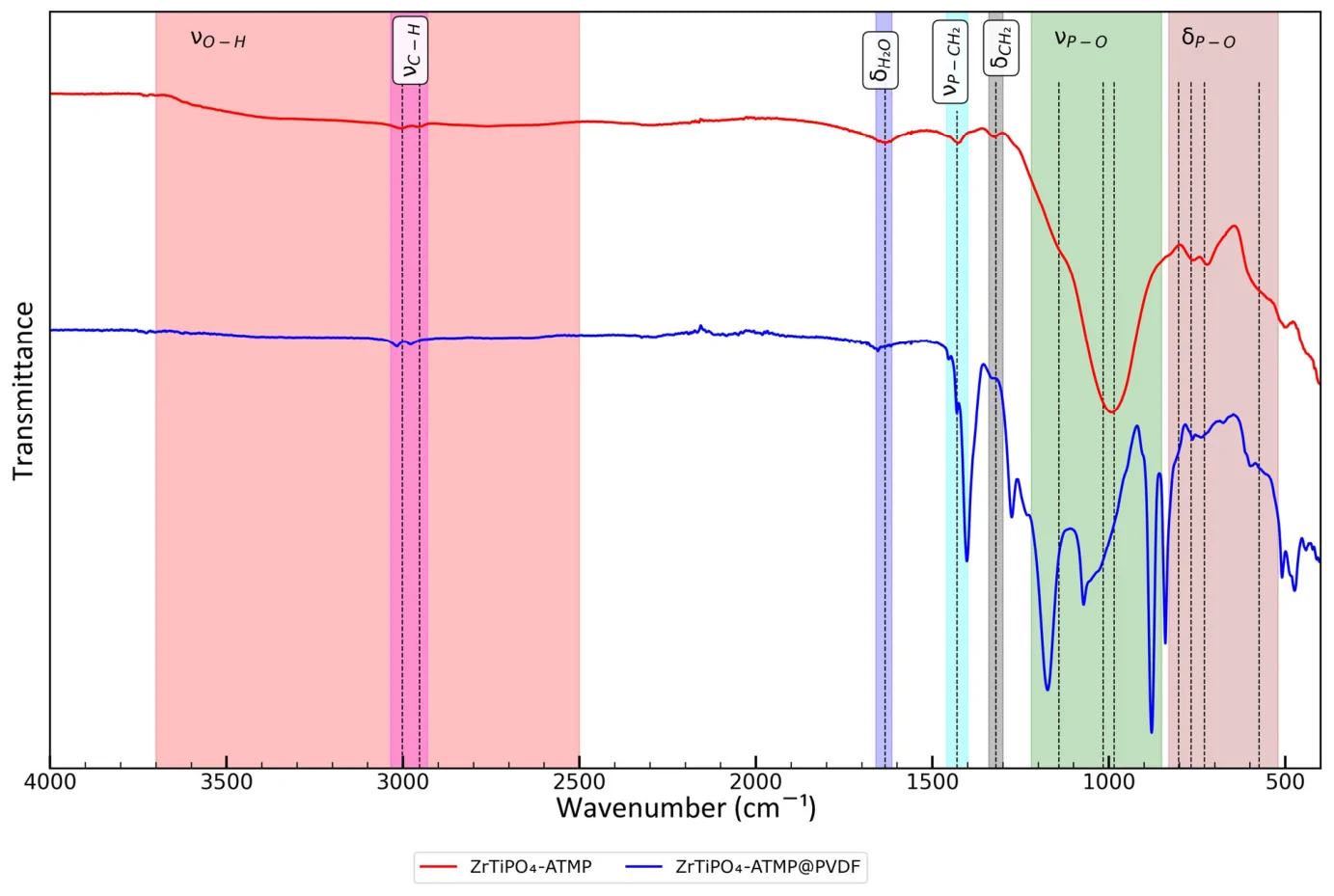
FTIR spectra of ZrTiPO_4_-ATMP powder and ZrTiPO_4_-ATMP@PVDF granules.

The FTIR spectra were analyzed to gain deeper insight into the structure of the obtained materials. The corresponding spectra are presented in Figure 5. The main bands associated with the phosphate-phosphonate material are marked, and their assignments to functional groups are also included in Figure 5 to support the discussion of the material’s structure.

The FTIR spectrum of the ZrTiPO_4_-ATMP material is very similar to those of the Zr-ATMP materials (particularly Zr-ATMP7) synthesized at a high P:Zr molar ratio in the reaction mixture discussed previously [36]. The only noticeable differences between the spectra of Zr-ATMP7 (see Figure 4 in [36]) and ZrTiPO_4_-ATMP are as follows: a slight shift and broadening of the ν_P-O_ bands toward lower wavenumbers (for ZrTiPO_4_-ATMP, the maximum of the main component is at 992 cm^−1^, whereas for Zr-ATMP7 it is at 1001 cm^−1^), and a shoulder band at 1142 cm^−1^, clearly visible in the Zr-ATMP7 spectrum, appears much weaker in the case of ZrTiPO_4_-ATMP. Such differences can be attributed to the presence of titanium phosphate moieties in the material’s structure, which influence the position of the band (for TiP5, the maximum of the main component of the band associated with ν_P–O_ is at 989 cm^−1^).

Comparing the spectra of ZrTiPO_4_-ATMP and ZrTiPO_4_-ATMP@PVDF, several additional sharp bands can be observed in the composite material. All these bands are predominantly associated with the β-phase (crystalline form I) of PVDF, with some contribution from the γ-phase (crystalline form III). The absence of a strong band at 615 cm^−1^ proves that the α-phase of PVDF is not present in the material [62]. The assignment of the bands to specific vibrations is as follows: 474 cm^−1^ w(CF_2_); 510 cm^−1^ δ(CF_2_); 839 cm^−1^ r(CH_2_)—ν_a_(CF_2_); 879 cm^−1^ ν_s_(CF_2_) + ν_s_(CC); 1074 cm^−1^ ν_a_(CC)—w(CF_2_) + w(CH_2_); 1172 cm^−1^ ν_a_(CF_2_)—r(CF_2_) + r(CH_2_); 1277 cm^−1^ ν_s_(CF_2_)—ν_s_(CC) + δ(CCC); 1399 cm^−1^ w(CH_2_)—ν_a_(CC); 1432 cm^−1^ δ(CH_2_); 2979 cm^−1^ ν_s_(CH_2_); and 3017 cm^−1^ ν_a_(CH_2_) [63].

**Figure 6:**
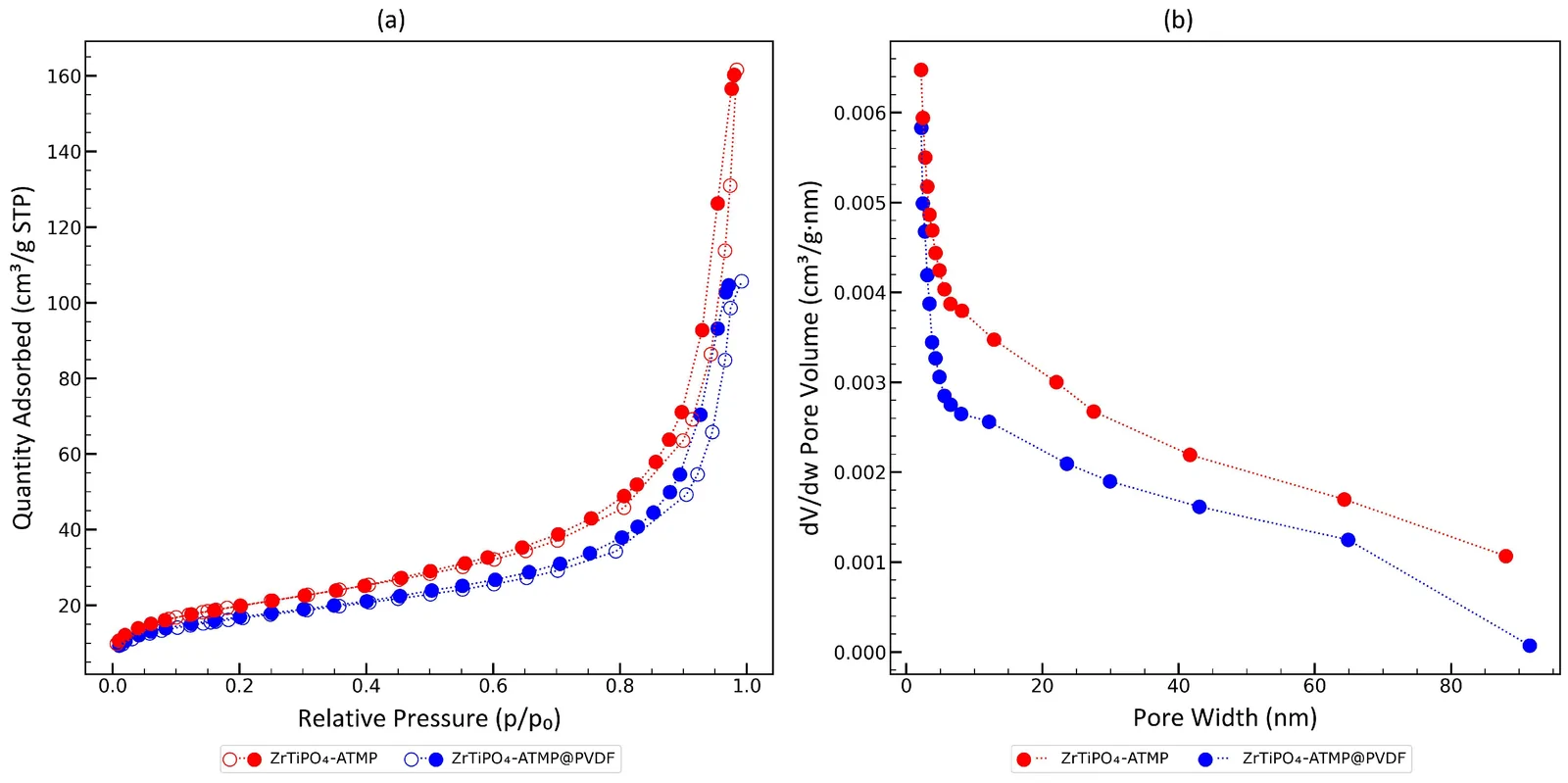
Nitrogen adsorption–desorption isotherms of (**a**) ZrTiPO_4_-ATMP powder and ZrTiPO_4_-ATMP@PVDF composite and (**b**) corresponding pore size distribution curves.

The N_2_ adsorption–desorption isotherms at 77 K for the ZrTiPO_4_-ATMP powder and its composite with PVDF are presented in Figure 6. Table 8 summarizes the textural properties of both materials.

A fundamental challenge in preparing such composites is achieving an appropriate combination of the binder and the active component so as to avoid the loss (or significant deterioration) of the properties of the original powder sorbent. The resulting nanocomposite beads exhibit favorable textural characteristics. The pristine ZrTiPO_4_-ATMP powder displayed a BET surface area of 71.43 m^2^/g and a BJH pore volume of 0.24 cm^3^/g, indicating a predominantly mesoporous structure with negligible microporosity. Following the incorporation of PVDF fibers as a binder, both the surface area (59.98 m^2^/g) and pore volume (0.15 cm^3^/g) decreased. This reduction can be attributed to the partial coverage of particle surfaces and pore blockage by the polymer matrix. The small hysteresis loop observed at higher relative pressures (>0.8) can suggest capillary condensation in the mesopores, and the isotherm can correspond to either type II or type IV according to the IUPAC classification. The amount of nitrogen adsorbed at p/p^0^ → 1 exceeds 100 cm^3^/g STP, suggesting a moderate specific surface area (neither very low nor extremely high). BJH analysis reveals a distinct maximum in the pore size range of a few nanometers (approximately 3–5 nm), indicating a predominance of mesopores. Simultaneously, the distribution is relatively broad, with a “tail” extending toward larger pores (10–50 nm) and even above 80 nm, suggesting the presence of both mesopores and macropores, which is consistent with the mercury intrusion porosimetry results. The low-temperature N_2_ adsorption results are consistent with SEM observations, where channels up to approximately 100 nm in width are visible.

The powder properties are typical for this class of materials. The BET surface area of 71.4 m^2^/g is similar to that reported for pure Zr-ATMP powder (62 m^2^/g) [36]. The t-plot micropore area is very small (3.38 m^2^/g), and likewise, the composite exhibits practically no micropores. Most of the surface area is contributed by mesopores, with BJH surface areas (1.7–300 nm) ranging from 57 to 69 m^2^/g (adsorption/desorption). The mesopore volume of the powder is 0.242 cm^3^/g, which is considerably higher than that of the composite (0.155 cm^3^/g), reflecting the agglomeration of powder grains by PVDF fibers. These results confirm that the composite retains mesoporosity, although with a reduced accessible surface area compared to the pristine powder.

**Figure 7:**
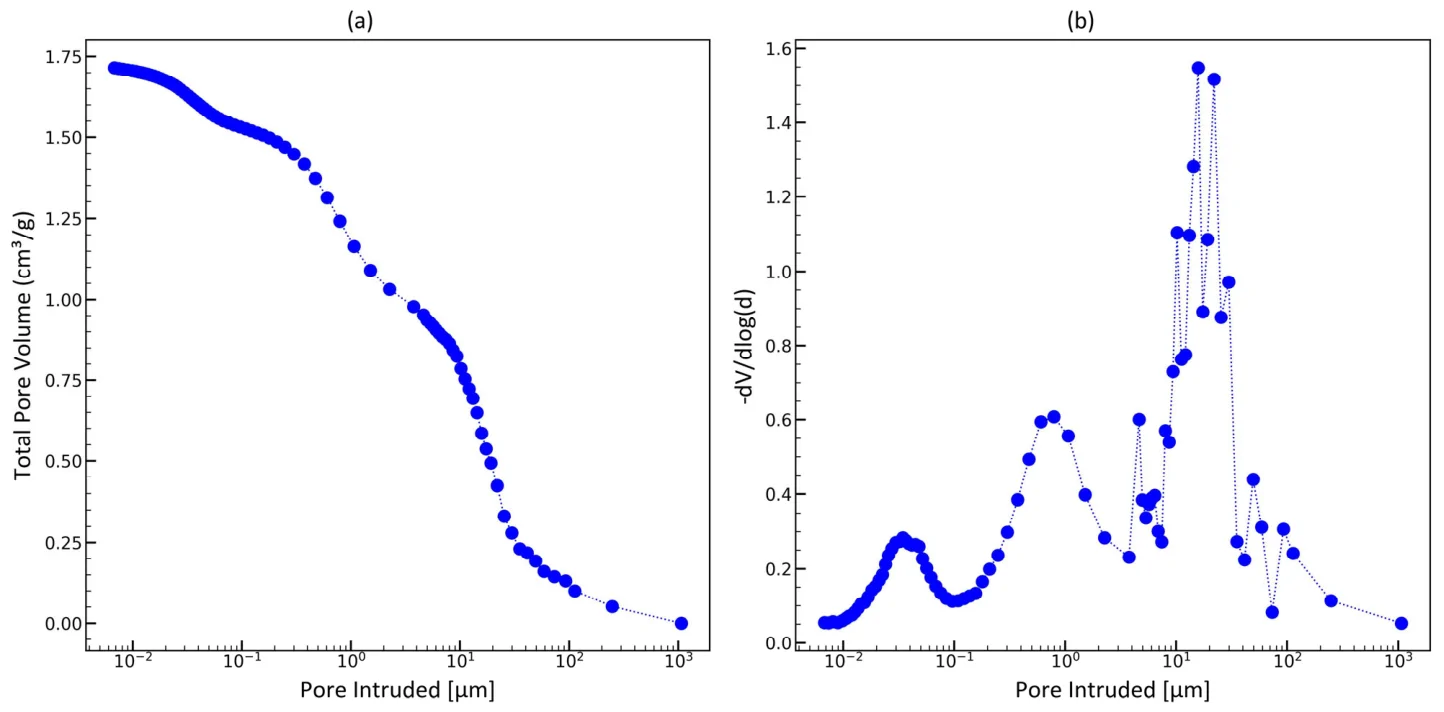
Mercury intrusion porosimetry results for ZrTiPO_4_-ATMP@PVDF: (**a**) cumulative pore volume curve and (**b**) pore size distribution.

Mercury intrusion porosimetry revealed a trimodal pore size distribution (Figure 7), with a dominant contribution in the macropore range, showing two maxima at approximately 20 μm and 0.8 μm. A much smaller peak was also observed in the mesopore region (10–100 nm), indicating the presence of both mesopores and macropores. The total porosity was estimated at 77.6%, and the cumulative pore volume reached approximately 1.71 cm^3^/g. Bulk and skeletal densities were estimated to be approximately 0.47 and 2.1 g/cm^3^, respectively. The presence of large macropores suggests a well-developed porous network, which may facilitate mass transport during sorption. The obtained results correspond well with the observations from SEM imaging and N_2_ adsorption studies.

### 3.4. Effect of pH on the Sorption Efficiency of ZrTiPO_4_-ATMP

Removal efficiency was determined in a series of samples with different pH adjustments, selected to achieve the desired pH at equilibrium. The tests were performed using a solution simulating the DLE eluate obtained according to Method 1, and the pH was adjusted using a 25% ammonia solution to avoid introducing additional metal ions. The results are presented in Table 9. It is evident that the sorption process, which proceeds via an ion-exchange mechanism, is strongly dependent on pH.

Carrying out the process under alkaline conditions results in significant lithium losses, whereas conducting it in such a way that the solution pH after contact with the sorbent drops below 4 leads to incomplete removal of calcium and magnesium. Therefore, the selection of the pH-adjusting agent dose (or precise control of reagent flow in a continuous system) has a crucial impact on the quality of the obtained eluate. The results presented in Table 9 also show that conducting the process at a slightly acidic pH (between 4 and 5) enables the complete removal of magnesium and calcium, as well as half of the sodium, without significant lithium losses. Moreover, the sorption selectivity sequence for the tested material can be expressed as Mg > Ca > Na > Li. Therefore, the developed sorbent meets the minimum requirements for its application in the purification of eluates obtained using the sorption-based DLE technology.

### 3.5. Sorption Kinetics of the ZrTiPO_4_-ATMP

For the sorbent to be used in a continuous process, a high sorption rate is required. Therefore, simple kinetic tests were performed by collecting samples after contact times ranging from 1 min to 24 h. The tests were performed at the optimal pH, and the results are presented in Table 10. According to the presented data, 95% or more of the equilibrium removal efficiency was achieved after 45 min of mixing for all analyzed elements. The fastest sorption was observed for calcium, and the slowest for sodium, while the lithium removal efficiency decreased during the analyzed period from 15% to 10%. The most probable explanation for this phenomenon is that, after the initial rapid sorption of lithium, desorption occurred due to the subsequent sorption of other elements more strongly bound to the sorbent. These results confirm the high sorption rate of the developed material.

### 3.6. Application of the ZrTiPO_4_-ATMP@PVDF for Purification of DLE Eluate

As shown in the previous sections, the developed sorbent can be used to purify diluted eluates obtained from DLE technology (i.e., eluates produced by a single lithium desorption step from saturated HTO). However, it is also worth testing whether such a sorbent can be applied to concentrated eluates obtained by multiple recirculations of the eluate in the DLE process. This case may be even more important, as the goal of the DLE process is to produce highly concentrated lithium solutions. It should be noted, however, that because of the high concentration of ions that need to be removed from such a solution, the consumption of the pH adjustment agent is also high. Moreover, the solution-to-sorbent ratio must be relatively low due to limitations associated with the sorption capacity. Nevertheless, removing elements from a more concentrated solution is easier because of the higher driving force for sorption.

Due to the fact that only a granulated sorbent can be used in the real process, such a composite was prepared as part of this work. Subsequent tests were performed using a composite sorbent material containing approximately 66% ZrTiPO_4_-ATMP. A concentrated 2 M LiOH solution was used as the pH adjustment agent (a source of OH^−^ ions). In a real DLE process, combined with the purification of lithium solution and production of LiOH using electrodialysis, a LiOH solution would be available on-site at the plant. Moreover, lithium ions would be retained in the eluate, so only hydroxide anions would be consumed in the sorption (ion-exchange) process.

The first series of static tests was performed at a constant liquid-to-sorbent ratio (L:S) of 4:1 (mL/g). This low ratio was chosen to evaluate the sorbent’s performance under optimal conditions. The variable parameter in this series was the dose of lithium hydroxide.

It should be noted that the total molar concentration of cations in the eluate is 0.63 M, which is relatively high. Of this amount, 25% corresponds to impurities that need to be removed. After conversion to H^+^ ion equivalents (which are released into the solution during ion exchange), the total concentration of cations is 0.69 eq/L. The total ion exchange capacity of the sorbent material is approximately 11 meq/g. Therefore, at the given L:S ratio, the total ion content in the eluate represents about 25% of the total ion exchange capacity of the sorbent material. Sorption tests were performed with varying additions of lithium hydroxide, ranging from 0 to 80% of theoretical hydroxyl ion demand, assuming complete sorption of all ions from the eluate. The removal efficiency for selected ions was determined and is presented in Table 11.

As can be seen, the addition of 80% of the stoichiometric OH^−^ ion demand allows for the removal of almost all calcium, magnesium, and potassium, and 80% of sodium, but with relatively high lithium losses (approximately 50%). Nevertheless, even under high salinity conditions, there is a clear preference for binding calcium, magnesium, potassium, and sodium ions over lithium ions, especially considering that the lithium concentration is higher than that of the other cations. With a much smaller dose of LiOH (25%), corresponding to the total amount of sodium, potassium, magnesium, and calcium ions in the solution portion, the removal of Ca, Mg, and K remains high (>90%), while the separation between Li and Na becomes more challenging. Moreover, at such a low L:S ratio, many functional groups of the sorbent are protonated and can also effectively bind lithium, leading to significant losses. Therefore, in the second series of experiments, the effect of increasing the L:S ratio on the removal efficiencies of individual elements was examined.

The second series of experiments was performed at a constant LiOH dose (25% of the OH^−^ equivalent of the total cation content in the solution) and at varying L:S ratios. The results are presented in Table 12.

Increasing the L:S ratio resulted in a decrease in lithium losses, which is attributed to the sorbent’s higher affinity for other ions. Nearly all calcium and magnesium were removed even at the highest L:S ratio, while the removal efficiency for potassium decreased with increasing L:S ratio. A major advantage of the material is its relatively high efficiency in removing sodium. The composition of the eluate after the purification process (L:S = 16) is presented in Table A1 in the Appendix. The higher lithium content in the eluate after purification, compared to the raw eluate, results from additional lithium ions introduced during pH adjustment with LiOH. As shown, the concentrate exhibited significantly improved quality following purification using the ZrTiPO_4_-ATMP@PVDF material.

Preliminary tests were also conducted to regenerate the sorbent using three different concentrations of hydrochloric acid (Table 13). The results demonstrate that effective desorption of the investigated ions was achieved using a 2 M HCl solution, with no significant operational issues observed. These experiments demonstrate the effectiveness of the regeneration step. However, they do not provide evidence of sorbent reusability, as repeated sorption–regeneration cycles were not investigated in the present study. The reusability of the sorbent will be investigated in future studies by evaluating its sorption performance over multiple consecutive sorption–regeneration cycles.

## 4. Conclusions

Lithium-rich eluates from the DLE process were successfully obtained using a hydrogen titanium oxide ion sieve and real oilfield brine. Two methods were applied, one of which yielded an eluate containing approximately 3600 mg/L Li. The resulting eluate was primarily contaminated with calcium and sodium (approximately 1200 mg/L Na and 2350 mg/L Ca).

In a preliminary screening study, twelve materials, including zirconium phosphates, titanium phosphates, and zirconium phosphonates, were evaluated for their ability to remove contaminants from DLE eluates. Based on the results obtained, the two best-performing materials, Zr-ATMP7 and TiP5, were selected. Zr-ATMP7 exhibited a very high affinity for calcium and magnesium ions, comparable to or even exceeding that of a commercially available resin (Puromet^TM^ MTS9500), while demonstrating no affinity for sodium ions. In contrast, TiP5 showed a high affinity for sodium ions.

For this reason, a novel material (ZrTiPO_4_-ATMP) containing both titanium and zirconium, as well as phosphonate and phosphate groups, was synthesized under mild conditions. This material exhibits affinity toward calcium, magnesium, potassium, and sodium ions, and this behavior was investigated using two types of DLE eluates.

Subsequently, a composite sorbent, ZrTiPO_4_-ATMP@PVDF, in granular form was prepared using PVDF as a binder. The sorption performance of the composite material was evaluated during the purification of an eluate obtained from real oilfield brine. Under optimal conditions, removal efficiencies exceeded 95% for calcium and magnesium and reached 75% and 60% for potassium and sodium, respectively, while lithium losses remained below 20%. Lithium losses can be further minimized by returning the regeneration solution to the sorption step.

To sum up, our study demonstrates that the ZrTiPO_4_-ATMP@PVDF sorbent exhibits promising properties for the removal of impurities from DLE eluates and may represent a viable alternative to conventional approaches such as precipitation, membrane separation, or recrystallization. To fully assess the practical applicability of ZrTiPO_4_-ATMP@PVDF under real process conditions, further studies are required, including more in-depth investigations of recyclability and long-term stability, testing in dynamic systems, and optimization of the material formulation. Nevertheless, the results obtained so far indicate that this class of materials holds significant potential for application in lithium refining processes.

## Figures and Tables

**Table 1:** Physicochemical properties of the raw oilfield brine.

Parameter	Value
TDS (g/L)	224
TSS (g/L)	0.28
Turbidity (FTU)	48
Mineral oil index (mg/L)	26
pH	6.37
Chloride (wt.%)	12.14

**Table 2:** Concentrations of the main metals in the obtained fractions.

Fraction	Li (mg/L)	Mg (mg/L)	Ca (mg/L)	Na (mg/L)	K (mg/L)
1	47	244	724	457	81
2	294	305	442	215	43
3	495	141	331	55	<10
4	273	78	163	45	<10

**Table 3:** Main parameters of the eluate obtained from the DLE process according to Method 2.

Cycle Number	q_Li_ * (mg/g)	R_Li_ (%)	Composition of Eluate				
			Li (mg/L)	Na (mg/L)	K (mg/L)	Mg (mg/L)	Ca (mg/L)
1	12.8	94.7	1796	467	21	16	1105
2	14.1	92.8	2865	959	43	28	1743
3	12.1	70.7	3319	1133	52	34	2154
4	11.7	65.7	3598	1166	62	38	2350

* q_Li_—Li working capacity.

**Table 4:** Degree of enrichment of individual metals in the eluate obtained from the DLE process according to Method 2.

Cycle Number	Degree of Enrichment *				
	Li	Na	K	Mg	Ca
1	7.4	0.009	0.007	0.014	0.025
2	11.9	0.018	0.014	0.024	0.040
3	13.7	0.021	0.017	0.029	0.049
4	14.9	0.022	0.021	0.032	0.054

* Degree of enrichment was calculated as follows: C_Me_ in eluate/C_Me_ in oilfield brine.

**Table 5:** Results of preliminary sorption tests.

Sorbent/Resin Name	Without pH Correction (pH ca. 2)				pH 8			
	R_Li_ (%)	R_Na_(%)	R_Mg_ (%)	R_Ca_ (%)	R_Li_ (%)	R_Na_ (%)	R_Mg_ (%)	R_Ca_ (%)
TiP3	2	47	<1	12	27	53	87	98
TiP4	2	46	<1	19	27	53	87	98
TiP5	3	73	<1	56	36	99	95	98
ZrP3	2	24	<1	16	28	56	43	76
ZrP4	<1	20	8	67	32	35	94	98
ZrP5	2	33	9	33	41	33	97	98
Zr-ATMP3	<1	9	<1	3	32	<1	91	97
Zr-ATMP4	6	25	13	69	47	<1	96	>99
Zr-ATMP5	5	10	16	66	44	<1	96	98
Zr-ATMP6	3	12	35	74	45	<1	98	99
Zr-ATMP7	4	6	27	85	52	<1	95	98
Zr-ATMP8	7	1	27	68	53	<1	95	98
MTS9500	20	-	36	56	26	-	90	96
TiP5 + Zr-ATMP7	5	40	21	77	39	71	94	98
ZrTiPO_4_-ATMP	2	37	58	91	25	79	>99	>99

**Table 6:** SEM-EDS analysis results of ZrTiPO_4_-ATMP powder and ZrTiPO_4_-ATMP@PVDF granules (surface and cross-section).

Element	Atomic %			
	ZrTiPO_4_-ATMP Powder		ZrTiPO_4_-ATMP@PVDF Granule	
	15 kV	25 kV	Surface (25 kV)	Cross-Section (25 kV)
C	11.5	26.7	33.3	24.9
O	49.7	51.2	10.1	22.4
P	26.0	10.1	4.7	6.3
Zr	7.9	4.3	0.2	1.2
N	0.3	7.2	0.1	4.7
Ti	4.6	0.5	0.4	0.8
F	0.0	0.0	51.2	39.7

**Table 7:** CHN analysis results of ZrTiPO_4_-ATMP powder and ZrTiPO_4_-ATMP@PVDF granules.

Sorbent Name	C (wt.%)	H (wt.%)	N (wt.%)
ZrTiPO_4_-ATMP	6.67	3.04	2.47
ZrTiPO_4_-ATMP@PVDF	17.94	3.10	2.22

**Table 8:** Textural properties of the ZrTiPO_4_-ATMP powder and ZrTiPO_4_-ATMP@PVDF composite.

Parameter	ZrTiPO_4_-ATMP	ZrTiPO_4_-ATMP@PVDF
BET surface area (m^2^/g)	71.43	59.99
t-plot micropore area (m^2^/g)	3.38	4.33
BJH adsorption cumulative surface area of pores between 1.7 nm and 300 nm in width (m^2^/g)	57.03	42.14
BJH adsorption cumulative volume of pores between 1.7 nm and 300 nm in width (cm^3^/g)	0.24	0.15
t-plot micropore volume (cm^3^/g)	0.0009	0.0013
BJH adsorption average pore width (4 V/A) (nm)	17.00	14.70

**Table 9:** Results of pH adjustment on the sorption efficiency of powdered ZrTiPO_4_-ATMP.

pH *	R_Li_ (%)	R_Na_ (%)	R_Mg_ (%)	R_Ca_ (%)
2	2	37	58	91
3	4	32	90	96
4	8	34	>99	98
5	10	50	>99	>99
7	25	73	>99	>99
8	25	79	>99	>99

* Measured at equilibrium.

**Table 10:** Results of the sorption kinetics of powdered ZrTiPO_4_-ATMP.

Time (min)	R_Li_ (%)	R_Na_ (%)	R_Mg_ (%)	R_Ca_ (%)
1	15	23	46	83
5	13	36	70	92
10	13	38	83	96
15	10	42	85	97
30	10	44	93	98
45	10	47	98	99
60	11	49	99	99
1440 *	10	50	>99	>99

* Equilibrium.

**Table 11:** Results of the sorption experiment with varying additions of lithium hydroxide.

LiOH Dose (%) ^a^	pH ^b^	R_Li_ (%)	R_Na_ (%)	R_K_ (%)	R_Mg_ (%)	R_Ca_ (%)
0	0.9	42	45	66	71	61
10	1.2	39	49	77	93	86
25	1.9	41	58	94	>99	97
40	5.8	45	69	>99	>99	>99
60	6.8	47	76	>99	>99	>99
80	7.4	48	80	>99	>99	>99

^a^ In the single experiment, 4 g of sorbent was mixed with 20 g of solution (total cation concentration in the solution was 0.69 eq/L). This amount of solution contained approximately 13.8 meq of cations. Therefore, the stoichiometric equivalent of OH^−^ ions required to neutralize the H^+^ ions released from the sorbent material during ion exchange was also 13.8 meq. For example, a 25% LiOH addition corresponds to 3.45 meq of OH^−^, which equals 3.45 mmol of LiOH. ^b^ at equilibrium.

**Table 12:** Results of the sorption experiment with varying L:S ratios.

L:S (mL/g)	pH *	R_Li_ (%)	R_Na_ (%)	R_K_ (%)	R_Mg_ (%)	R_Ca_ (%)
4	1.9	41	58	94	>99	97
8	5.2	40	72	91	99	98
12	5.8	26	64	84	98	97
16	6.5	18	60	75	96	97
20	6.5	16	56	67	94	96

* At equilibrium.

**Table 13:** Results of the regeneration experiment.

HCl Concentration	R_Li_ (%)	R_Na_ (%)	R_K_ (%)	R_Mg_ (%)	R_Ca_ (%)
1 M	>95	>95	68	90	84
2 M	>95	>95	>95	>95	>95
4 M	>95	>95	>95	>95	>95

## Data Availability

The original contributions presented in this study are included in the article. Further inquiries can be directed to the corresponding author.

## Abbreviations

The following abbreviations are used in this manuscript:

DLE	direct lithium extraction
LiAl-LDHs	lithium-aluminum layered double hydroxides
LMO	lithium manganese oxide
LTO	lithium titanium oxide
HTO	hydrogen titanium oxide
DMF	dimethylformamide
PVDF	poly(vinylidene fluoride)
ATMP	aminotris(methylenephosphonic acid)
AAS	atomic absorption spectrometry
XRD	X-ray diffraction
FTIR	Fourier transform infrared
ATR	attenuated total reflection
SEM	scanning electron microscopy
EDS	energy dispersive X-ray spectroscopy
BET	Brunauer–Emmett–Teller
BJH	Barrett–Joyner–Halenda
DFT	density functional theory
MIP	mercury intrusion porosimetry
ICP-MS	inductively coupled plasma mass spectrometry
TDS	total dissolved solids
TSS	total suspended solids

## Appendix A

**Table A1:** Comparison of the compositions of raw oilfield brine, lithium-rich eluate from the DLE process (obtained via Method 2), and the eluate after treatment.

Analyte	Raw Oilfield Brine	Lithium-Rich Eluate	Eluate After Treatment
Na	50.8 [g/L]	1166 [mg/L]	213 [mg/L]
Ca	26.2 [g/L]	2350 [mg/L]	6 [mg/L]
K [mg/L]	2757	38	<1
Mg [mg/L]	1175	62	<1
Sr [mg/L]	927	139	<1
B [mg/L]	755	25	21
Li [mg/L]	241	3598	3800
Fe [mg/L]	55	<1	<1
Rb [mg/L]	7.8	<0.05	<0.05
Ba [mg/L]	4.5	28.7	<1
Cs [mg/L]	2.4	0.4	0.4
Al [mg/L]	<10	<10	<10
V [μg/L]	715	129	113
Zr [μg/L]	477	479	600
Mn [μg/L]	430	3297	<300
Ni [μg/L]	320	<200	<200
Be [μg/L]	<400	<400	<400
Ag [μg/L]	<300	<300	<300
Co [μg/L]	<50	<50	<50
Cd [μg/L]	<100	<100	<100
Pb [μg/L]	<600	<600	<600
Ti [μg/L]	<900	2351	1254
